# Selective effects of estradiol on human corneal endothelial cells

**DOI:** 10.1038/s41598-023-42290-z

**Published:** 2023-09-15

**Authors:** Seoyoung Han, Christian Mueller, Caitlin Wuebbolt, Sean Kilcullen, Varinda Nayyar, Brayan Calle Gonzalez, Ali Mahdavi Fard, Jamie C. Floss, Michael J. Morales, Sangita P. Patel

**Affiliations:** 1 Jacobs School of Medicine and Biomedical Sciences, University at Buffalo, State University of New York, Buffalo, NY USA; 2 Research Service, Veterans Administration Western New York Healthcare System, Buffalo, NY USA; 3 Department of Ophthalmology, Ross Eye Institute, Jacobs School of Medicine and Biomedical Sciences, University at Buffalo, State University of New York, Buffalo, NY USA; 4 Department of Physiology and Biophysics, Jacobs School of Medicine and Biomedical Sciences, University at Buffalo, State University of New York, Buffalo, NY USA; 5 Ophthalmology Service, Veterans Administration Western New York Healthcare System, Buffalo, NY USA

**Keywords:** Corneal diseases, Cell biology, Physiology

## Abstract

In Fuchs endothelial corneal dystrophy (FECD), mitochondrial and oxidative stresses in corneal endothelial cells (HCEnCs) contribute to cell demise and disease progression. FECD is more common in women than men, but the basis for this observation is poorly understood. To understand the sex disparity in FECD prevalence, we studied the effects of the sex hormone 17-β estradiol (E2) on growth, oxidative stress, and metabolism in primary cultures of HCEnCs grown under physiologic ([O_2_]_2.5_) and hyperoxic ([O_2_]_A_) conditions. We hypothesized that E2 would counter the damage of oxidative stress generated at [O_2_]_A_. HCEnCs were treated with or without E2 (10 nM) for 7–10 days under both conditions. Treatment with E2 did not significantly alter HCEnC density, viability, ROS levels, oxidative DNA damage, oxygen consumption rates, or extracellular acidification rates in either condition. E2 disrupted mitochondrial morphology in HCEnCs solely from female donors in the [O_2_]_A_ condition. ATP levels were significantly higher at [O_2_]_2.5_ than at [O_2_]_A_ in HCEnCs from female donors only, but were not affected by E2. Our findings demonstrate the resilience of HCEnCs against hyperoxic stress. The effects of hyperoxia and E2 on HCEnCs from female donors suggest cell sex-specific mechanisms of toxicity and hormonal influences.

## Introduction

Fuchs endothelial corneal dystrophy (FECD), the leading indication for corneal transplantation in the United States, is twice as common in women than in men, but the underlying pathophysiologic mechanisms for this disparity are unknown^1–4^. Genetic and extrinsic factors contribute to the development of the common adult-onset form of FECD. A trinucleotide repeat expansion in the *TCF4* gene is commonly associated with FECD in those of European ancestry; however, not all individuals with this expansion demonstrate the disease phenotype, and genetics do not explain why FECD is more common in women^5,6^. Extrinsic stressors thus play an important role in the development of FECD.

In FECD, the nonproliferative corneal endothelial cells on the posterior cornea experience oxidative stress, mitochondrial stress, and metabolic stress potentiated by elevated oxygen levels in the anterior chamber of the eye and by ultraviolet light exposure^7,8^. These extrinsic stressors contribute to basement membrane abnormalities (guttae in Descemet's membrane) and corneal endothelial cell death. In mice, ultraviolet-A light exposure is sufficient to generate the FECD phenotype, with guttae and corneal endothelial cell loss and greater disease burden in female mice than in male mice^9^.

We want to understand why FECD is more common in women than in men. We focused our study on 17-β estradiol (**E2**), the most potent circulating estrogen in humans. E2 levels in premenopausal women are higher than in men; however, postmenopausal women have lower levels than men^10^. The changes around menopause have been implicated in cardiovascular disease^11^, ischemic stroke^12^, and ocular surface disease^13^. Prior epidemiologic investigations suggest that the peri/postmenopausal age range (~ 50–59 years), a common age bracket for diagnosis of FECD, has a disproportionate burden of FECD in women than in men compared to that for other age brackets, implicating factors associated with the menopausal transition in FECD^14,15^. Prior laboratory research suggests that estrogen metabolites can harm corneal endothelial cells by forming genotoxic estrogen-DNA adducts leading to DNA damage and apoptotic cell death^16^. However, in studies of cellular and molecular mechanisms of neuronal and cardiovascular diseases, estrogen has shown protective effects^17–21^.

The purpose of our study was to examine the effects of E2 on human corneal endothelial cells (**HCEnCs**) to explore in vitro if E2 is a factor contributing to the greater prevalence of FECD in women than in men. We used a chronic hyperoxic stress model to test the effects of E2 on growth, oxidative stress, and metabolism in primary HCEnC cultures.

## Methods

### Corneal tissues

The use of human tissues for these studies was approved by the University at Buffalo and VA Western NY Healthcare System Institutional Review Boards, and all research protocols were approved by the VA Western NY Research and Development Committee. Human subjects provided written informed consent and protocols were performed in accordance with the Declaration of Helsinki. Human cadaver corneal tissues for these studies were obtained with permission from the Anatomical Gift Program at the University at Buffalo with consent for research use of the human tissues obtained by the Anatomical Gift Program. Human surgical FECD samples from endothelial keratoplasty were obtained from patients with visually significant FECD after obtaining written informed consent. The age and sex of all tissue donors are listed in Table 1. Patient enrollment and all experiments were performed between 2016 and 2023.

**Table 1:** Donor tissue information.

Donor	Type	Age (yr)	Sex	Lens status^a^	Assay
1	FECD	58	F	pcIOL	RT-qPCR
2	FECD	61	F	Phakic	RT-qPCR
3	FECD	77	F	Phakic	RT-qPCR
4	Healthy	84	M	Phakic	RT-qPCR
5	Healthy	67	M	Phakic	RT-qPCR
6	Healthy	76	F	Phakic	RT-qPCR
7	Healthy	96	F	pcIOL	RT-qPCR
8	Healthy	90	M	Unknown	Western blotting, GPER
9	Healthy	71	M	Unknown	Western blotting, ERβ
10	Healthy	77	M	Unknown	Western blotting, ERβ
11	Healthy	76	M	Unknown	Immunofluorescence, ERβ
12	Healthy	39	M	Phakic	Immunofluorescence, ERβ
13	Healthy	74	F	Unknown	Immunofluorescence, ERβ
14L	Healthy	71	M	pcIOL	Cell viability
14R	Healthy	71	M	pcIOL	Cell viability
15	Healthy	69	M	Phakic	Cell viability
16	Healthy	83	M	pcIOL	Cell viability
17	Healthy	91	M	pcIOL	Cell viability
18	Healthy	74	M	Phakic	ROS
19	Healthy	91	M	pcIOL	ROS
20	Healthy	94	F	pcIOL	ROS
21	Healthy	53	M	Phakic	ROS
22	Healthy	71	F	Unknown	ROS
23	Healthy	82	M	pcIOL	ROS
24	Healthy	88	F	pcIOL	ROS
25	Healthy	63	F	Phakic	ROS
26L	Healthy	61	F	Phakic	ROS
26R	Healthy	61	F	Phakic	ATP
27R	Healthy	83	F	Phakic	MitoTracker + 8-oxo-dG
27L	Healthy	83	F	Phakic	MitoTracker + 8-oxo-dG
28L	Healthy	88	F	pcIOL	MitoTracker + 8-oxo-dG
29R	Healthy	65	F	Phakic	MitoTracker + 8-oxo-dG
30L	Healthy	86	F	pcIOL	MitoTracker + 8-oxo-dG
31L	Healthy	72	M	pcIOL	MitoTracker + 8-oxo-dG
32L	Healthy	80	M	pcIOL	MitoTracker + 8-oxo-dG
33R	Healthy	77	M	Phakic	MitoTracker + 8-oxo-dG
33L	Healthy	77	M	Phakic	MitoTracker + 8-oxo-dG
34L	Healthy	74	M	Phakic	MitoTracker + 8-oxo-dG
35R	Healthy	97	F	pcIOL	ATP
36L	Healthy	71	F	Phakic	ATP
37L	Healthy	63	F	pcIOL	ATP
38R	Healthy	87	M	pcIOL	ATP
38L	Healthy	87	M	pcIOL	ATP
39R	Healthy	67	M	pcIOL	ATP
39L	Healthy	67	M	pcIOL	ATP
40R	Healthy	91	M	pcIOL	Seahorse
41	Healthy	79	F	pcIOL	Seahorse
42	Healthy	67	F	Phakic	Seahorse
43	Healthy	69	M	pcIOL	Seahorse
44	Healthy	62	F	Phakic	Seahorse
45	Healthy	77	M	Phakic	Seahorse
46	Healthy	71	M`	Phakic	Western blotting, ERβ
47	Healthy	74	M	pcIOL	Western blotting, ERβ

^a^pclOL, posterior chamber intraocular lens.

### Cell cultures

All studies of HCEnCs were performed with primary, P0, corneal endothelial cell cultures using previously published protocols^22^. Cultures were initiated from human corneas obtained within 24 h after death. Corneoscleral buttons were dissected from the eyes and were examined for the presence of central guttae. Corneas with guttae were excluded from the cell culture experiments. Descemet's membrane with adherent endothelial cells was stripped from the corneoscleral buttons and incubated overnight at 37 °C in minimal medium (human endothelial SFM, Gibco, cat# 11111-044) with 2% charcoal stripped fetal bovine serum (FBS) and 1× antibiotic–antimycotic (Gibco, cat# 15240-062). Cells were dissociated with 0.02% EDTA (Sigma-Aldrich, cat# 45-E8008). Cells were resuspended in growth medium (Opti-MEM 1 [Gibco], 8% FBS, 20 µg/mL ascorbic acid, 200 mg/L calcium chloride, 1× antibiotic–antimycotic, 100 µg/mL pituitary extract, 5 ng/mL epidermal growth factor, and 50 µg/mL gentamicin) and distributed on FNC-coated (Athena, cat# 0407) plates or coverslips according to the experimental plan.

Cultures were incubated at 37 °C in standard room air incubators ([O_2_]_A_: room air + 5% CO_2_, humidified incubator) or at 2.5% O_2_ in a modular incubation chamber ([O_2_]_2.5_: 2.5% O_2_ + 5% CO_2_ + balance N_2_, humidified; Billups-Rothenberg, Inc., Del Mar, CA, USA). HCEnCs were expanded in growth medium to confluence and then matured to a stable phenotype in minimal medium. Charcoal-stripped FBS was used in the minimal medium to remove endogenous steroid hormones from the serum. For experiments testing estradiol exposure, E2 (stock concentrations selected for preparation in dimethyl sulfoxide with 500-fold dilution into culture medium to achieve final desired concentrations; Sigma- Aldrich, cat# E8875) was added for 7–10 days in the maturation phase of the HCEnC culture.

MCF-7 and PC3 cells were provided by colleagues who purchased them from American Type Culture Collection^23,24^. MCF-7 cells were cultured in DMEM (Corning, cat# 10-017-CV) + 10% FBS + 1× antibiotic–antimycotic and used at passages 16–26. PC3 cells were grown in RPMI 1640 medium (Corning, cat# 10-040-CV) with 10% FBS and 1 × antibiotic–antimycotic and used at passages 12–26. Cells were maintained in T25 flasks in a 5% CO_2_, humidified 37 °C tissue culture incubator.

### RT-qPCR

Transcript levels for estrogen receptor (**ER**) α (**ERα**), estrogen receptor β (**ERβ**), and G protein-coupled estrogen receptor (**GPER**) 1 were measured by reverse transcription-real time quantitative polymerase chain reaction (RT-qPCR). RNA was purified from individual corneas by using an RNeasy Plus universal kit (Qiagen). cDNA was synthesized using the iScript Advanced cDNA synthesis kit for RT-qPCR (Bio-Rad Laboratories) with 2.0–20 ng input corneal RNA according to the manufacturer's protocol. RT-qPCR was performed using the Bio-Rad SsoAdvanced universal SYBR green kit with 2 μL cDNA (10% total volume) in a 96-well plate. Reactions were performed on a Bio-Rad C1000 thermal cycler equipped with a CFX96 real-time system running CFX Manager software (version 3.1). Assays were run in triplicates and included controls for genomic DNA contamination, PCR efficiency, and reverse transcription. Primers were designed and validated by the manufacturer (PrimePCR SYBR green assay, Bio-Rad Laboratories) and are listed in Supplementary Table 1. The quantification cycles were calculated by CFX Manager. Transcript levels for ERα, ERβ, and GPER in normal and FECD samples were normalized to the level of ERα in normal samples. The mean from the data were compared with *t* tests assuming equal variance.

### Western blotting

Western blotting was performed similarly to published protocols^22^. Protein was isolated from confluent PC3 and MCF7 cultures or from human samples, from which Descemet’s membrane-endothelium complex or retinal pigment epithelium was stripped, stored at − 80 °C, and thawed in lysis buffer. Samples were disrupted mechanically with a pestle in lysis buffer (50 mM Tris [pH 7.4], 250 mM NaCl, 2 mM ethylenediaminetetraacetic acid [pH 8.0], 10% protease inhibitor cocktail [Sigma-Aldrich], and 10% Triton X-100). Protein concentration was measured with the Pierce BCA kit (Thermo Fisher Scientific), and 10 μg protein was loaded per lane on Mini-PROTEAN TGX precast gels (Bio-Rad Laboratories). After protein separation, samples were transferred to PVDF membranes (Bio-Rad tank blotting system), blocked with 10% goat serum, and incubated with primary antibodies (mouse anti-ERβ antibody: cat# sc-390243, lot# K1814 [Santa Cruz Biotechnology], used at 1:250 dilution in blocking buffer; rabbit anti-ERβ: cat# sc-8974, lot# D0615 [Santa Cruz Biotechnology], used at 1:250 dilution in blocking buffer; rabbit anti-GPER1 antibody: cat# HPA027052, lot# D118286 [Sigma-Aldrich], used at 1:500 diluted in blocking buffer) overnight at 4 °C. After washing three times in TBST, the membranes were incubated for 1 h at room temperature with secondary antibodies (goat anti-rabbit IgG-alkaline phosphatase, cat# A9919 [Sigma-Aldrich]; goat anti-mouse IgG-alkaline phosphatase, cat# A4312, [Sigma-Aldrich]; 1:3,000). Signals were developed with ECF substrate (Cytiva Amersham) and imaged with the ChemiDoc MP Imaging System (Bio-Rad Laboratories).

### ER immunofluorescence localization

Descemet's membrane-endothelium complex was stripped from fresh human corneas and fixed immediately in 3% paraformaldehyde at 4 °C. The tissue was rinsed three times with PBS, permeabilized with 0.1% Triton X-100 for 5 min, and washed two more times with PBS. After blocking nonspecific antibody binding with 10% goat serum for 30 min at room temperature, the samples were incubated for 2 h with mouse anti-ERβ (1:100) at room temperature. The sample was washed with PBS three times and incubated at room temperature for 1 h with goat anti-mouse IgG conjugated to Alexa Fluor 568 (1:250, Invitrogen, cat# A11011). The sample washed three times with PBS and placed carefully in a single layer onto a microscope slide containing a drop of Vectashield with DAPI (Vector Laboratories). Samples were imaged by laser confocal microscopy (Leica TCS SPEII DMI4000).

### ER agonist experiments in PC3 cells and HCEnCs

PC3 cells between passages 12–15 were collected at 70% confluency and seeded at 30,000 cells per well in a 24-well plate with culture medium containing charcoal-stripped FBS. After 24 h, each well was treated with 1, 10, or 100 nM E2. Similarly, other wells were treated with 0.1, 1.0, or 10 µM G1 GPER agonist (Azano Biotech, Albuquerque, NM) dissolved in culture medium. Cells were incubated for 3 days (without medium change) and then lifted by trypsinization and stained with trypan blue; viable cells from each well were counted with a hemocytometer. Data from four experiments were averaged and compared with *t* tests assuming equal variance.

For HCEnCs, cells from one cornea were distributed into 16 wells (eight for [O_2_]_A_ and eight for [O_2_]_2.5_) of 96-well plates. Once cells reached confluence, the medium was changed to minimal medium (with charcoal-stripped FBS) with or without E2 or G1 (two wells for each condition). After incubating for 7–10 days, cell nuclei were stained with DAPI and counted to determine the total cell count. The means from the data were compared by ANOVA with significance set at a *p* value of < 0.05.

### Cell viability assays

HCEnCs were cultured in two 96-well plates, with cells from one cornea distributed into 16 wells (eight for [O_2_]_A_ and eight for [O_2_]_2.5_). The cells were grown in growth medium until confluence and then matured in minimal medium. During the maturation phase, cells were grown under different conditions (two wells per condition): control, 1 μM GPER agonist (AZ00004-G1, Azano Biotech), or 10 nM E2. Two wells with minimal medium without cells were used as a negative control. Cell viability was measured using the RealTime Glo cell viability assay (Promega). The MT viability substrate and NanoLuc enzyme (1:2000 dilution) were added to each well, and luminescence was measured with a Synergy HT plate reader (BioTek). The nuclei of cells were then stained with DAPI and counted. Luminescence readings were normalized to the cell count per well. The means from the data were compared by ANOVA with significance at a *p* value of < 0.05.

### Measurements of cellular ROS

Primary HCEnCs from one cornea were distributed into four wells (two for [O_2_]_A_ and two for [O_2_]_2.5_) of 24-well plates. Cells were fed with growth medium until confluence and then matured for 7–10 days in minimal medium with or without 10 nM E2. Supernatants from each plate were removed and HCEnCs were immediately lysed with 0.5% Triton X-100 in PBS. Lysed cells were transferred to microcentrifuge tubes, incubated on ice for 20 min, and centrifuged at 4 °C for 20 min to pellet cell debris; the supernatant was collected for analysis. Reactive oxygen species (ROS) levels were analyzed with the In Vitro ROS/RNS assay (Cell Biolabs San Diego, CA). Briefly, 35 μL of cell lysate mixed with 15 μL PBS was added to wells of a 96-well plate; 100 μL of dichlorodihydrofluorescien solution (prepared according to the manufacturer's instructions) was added to each well and incubated for 30 min while protected from light. Fluorescence was measured for 5 min with a plate reader (Synergy HT, BioTek) (excitation, 480 nm; emission, 530 nm). Fluorescence measurements from cell lysates were compared to those for a H_2_O_2_ standard curve and normalized to total protein concentration in each sample. The protein concentration of the lysate was determined with the Pierce BCA kit. The means of the data were compared by ANOVA with significance at a *p* value of < 0.05.

### MitoTracker and 8-oxo-dG immunofluorescence staining

HCEnCs were seeded in 48-well plates (one cornea per 8 wells) with round #1.5 glass coverslips. Cells were grown to confluence in growth medium and then switched to minimal medium with or without 10 nM E2 for 1–2 weeks prior to an 8-hydroxy-2'-deoxyguanosine (8-oxo-dG) assay. Briefly, 48 and 24 h before staining, 100 μM H_2_O_2_ was added to the culture medium in the desired wells. At the time of assay, HCEnCs were incubated in 200 nM MitoTracker Red CMXRos (Invitrogen, cat# M5712) by adding 1.0 μL of a 50 μM stock to each well containing 250 µL of minimal medium for 15 min at 37 °C. Cells were then fixed with 1:1 methanol:acetone for 20 min at − 20 °C. Methanol:acetone was then removed, and samples were allowed to air dry before undergoing sequential washes with PBS and 35%, 50%, and 75% ethanol in H_2_O for 3 min each. The cells were incubated for 4 min in 0.15 N sodium hydroxide prepared in 70% ethanol to denature the DNA and then fixed in 3.7% paraformaldehyde in 70% ethanol for 2 min. Samples were then washed with 50% ethanol, 35% ethanol, and PBS for 2 min each. Cells were permeabilized in 0.1% Triton X-100 for 5 min and washed twice with PBS. Nonspecific antibody binding was blocked with 10% normal goat serum in PBS for 30 min at room temperature, and then the samples were incubated for 1 h with anti-8-oxo-dG antibody (1:400 in 1% goat serum; R&D Systems, cat# 4354-MC-050, lot# P275856). After three washes in PBS, the cells were incubated for 1 h at room temperature in secondary antibody (goat anti-mouse IgG conjugated to Alexa Fluor 488) diluted 1:1000 in PBS containing 1% goat serum. Cells were again washed three times with PBS and then once with deionized water. The samples were then mounted with Vectashield with DAPI and imaged by fluorescence microscopy with a Keyence scanner (Keyence BZ-X810; Keyence, Osaka, Japan) at identical exposure and acquisition settings. From the digital images, a minimum of 50 cells from each sample well were analyzed. The morphology of mitochondria in each cell was graded by observers blind to the conditions as diffuse (normal), intermediate, or fragmented (abnormal)^25^; 8-oxo-dG was graded as positive or negative by nuclear stain. The means from the data were analyzed by ANOVA with significance at a *p* value of < 0.05. Findings of significance were explored with Tukey's post hoc test.

### ATP assay

HCEnCs from each cornea were distributed into four wells (two for [O_2_]_A_ and two for [O_2_]_2.5_) in 24-well plates and grown to confluence in growth medium before being treated in minimal medium with or without 10 nM E2 for 7–10 days. ATP concentrations were measured using a luminescent ATP detection kit (Abcam) according to the manufacturer's instructions. First, 50 μL of kit detergent was added to each well, and the plate was sealed and placed on an orbital shaker at 600–700 rpm for 5 min to lyse cells and stabilize ATP. The cell lysates were transferred to a 96-well plate; 50 μL of substrate solution was added to each well, and the plate was shaken again on the orbital shaker for 5 min. The plate was dark adapted for 10 min before luminescence was measured on a plate reader (Synergy HT). Raw luminescence values were compared to those for a standard curve run in parallel with the assay in order to calculate the ATP concentration for each well. Means for the ATP concentrations measured from each condition were compared by ANOVA and Tukey’s tests with significance set at a *p* value of < 0.05.

### Seahorse assay

A Seahorse XFe24 analyzer (Agilent Technologies) was used to measure O_2_ consumption rates (**OCRs**; reflective of mitochondrial respiration) and extracellular acidification rates (**ECARs**; reflective of glycolysis). Primary HCEnCs were dissociated and seeded into FNC-coated Seahorse XFe24 culture plates. Cells from one donor cornea were seeded into 10 wells (five for [O_2_]_A_ and five for [O_2_]_2.5_). Cells were expanded until confluence in growth medium and then matured for 7–10 days in minimal medium. Half of the samples had minimal medium with 10 nM E2; the other half served as control (without E2). The Seahorse XFe24 sensor cartridges and drugs were prepared according to the manufacturer's protocols as previously described by our laboratory^22^. The assay medium was Seahorse XF DMEM (with 1.2 mM glutamine, 7.0 mM glucose, and 0.45 mM pyruvate). OCR and ECAR measurements were taken at baseline and after additions of oligomycin (1 μM), FCCP (1.5 μM), rotenone/antimycin A (0.5 μM), and 2-DG (50 mM; all drugs were purchased from Sigma-Aldrich). The cells were then fixed with 1:1 methanol:acetone at − 20 °C and nuclei were stained with DAPI and counted to normalize OCR and ECAR measurements to cell density. Trends in means from the data after each Seahorse assay drug addition were compared between control and E2 groups at [O_2_]_A_ and [O_2_]_2.5_ using two-tailed *t* tests.

## Results

### ERs in human corneal endothelium

**Figure 1:**
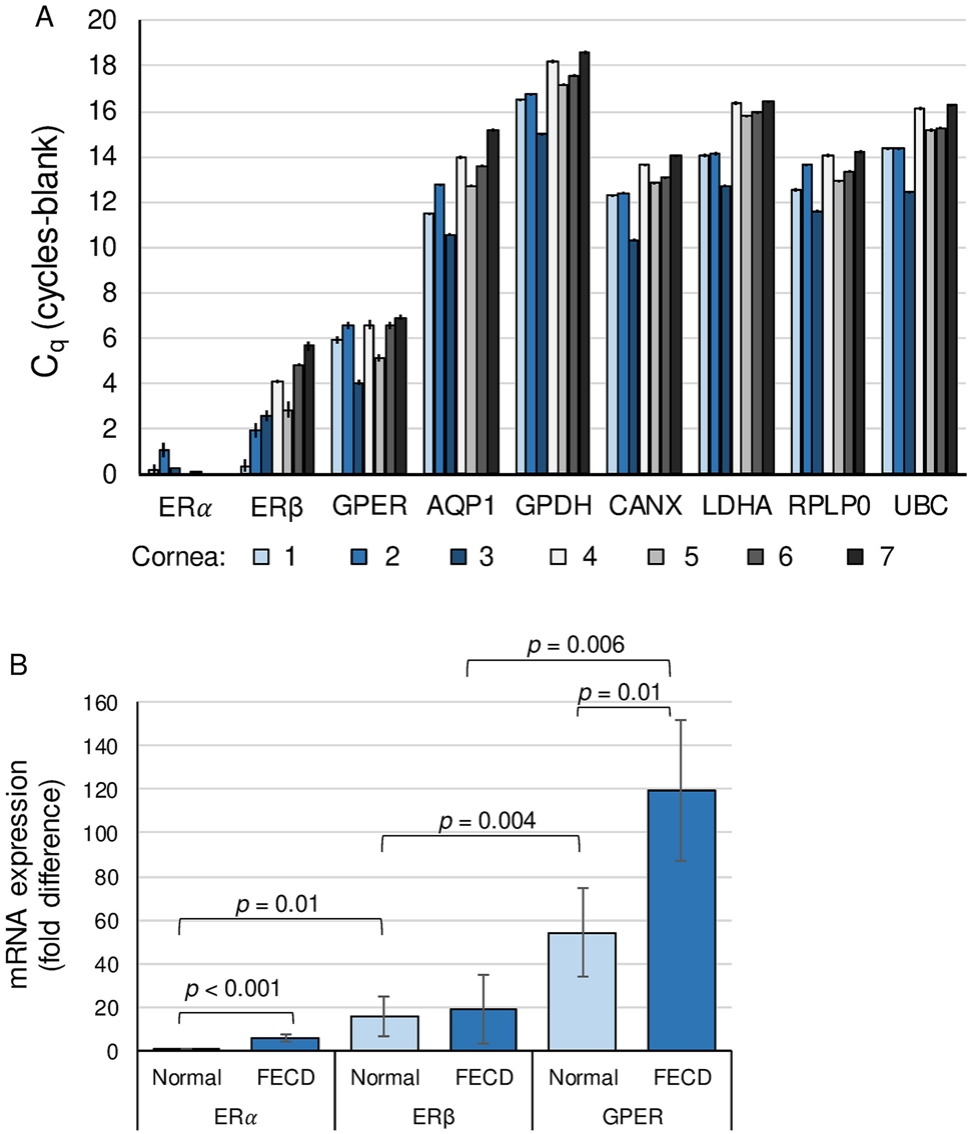
ER gene expression in human corneal endothelium. RT-qPCR was performed to measure mRNA expression for *ESR1* (ERα), *ESR2* *(ERβ), GPER,* and housekeeping/control genes (*AQP1* [aquaporin 1], *GAPDH* [glyceraldehyde-3-phosphate dehydrogenase], *CANX* [calnexin], *LDHA* [lactate dehydrogenase A], *RPLP0* [ribosomal protein lateral stalk subunit P0], and *UBC* [ubiquitin C]) in human corneal endothelium from non-diseased donors (*n* = 4, corneas #4–7 in Table 1) and donors with FECD (*n* = 3, corneas #1–3). (**A**) Data presented are the means from three reverse transcription reactions ± standard deviations. (**B**) Fold-changes in estrogen receptor expression compared to ERα expression in non-diseased corneal endothelium.

To determine if estrogen affects the corneal endothelium by canonical ER signaling pathways, we investigated the expression of ERs in native human corneal endothelium from males and females with and without FECD. RT-qPCR revealed in normal and FECD corneal endothelium that *GPER* was most abundantly expressed followed by *ESR2* (coding for ERβ) then *ESR1* (coding for ERα; Fig. 1A). In corneal endothelium from donors without FECD, ERβ expression was 15.7-fold greater, and GPER was 54.2-fold greater than ERα (Fig. 1B). ERα and GPER had significantly higher expression in corneal endothelium from FECD samples compared to normal.

**Figure 2:**
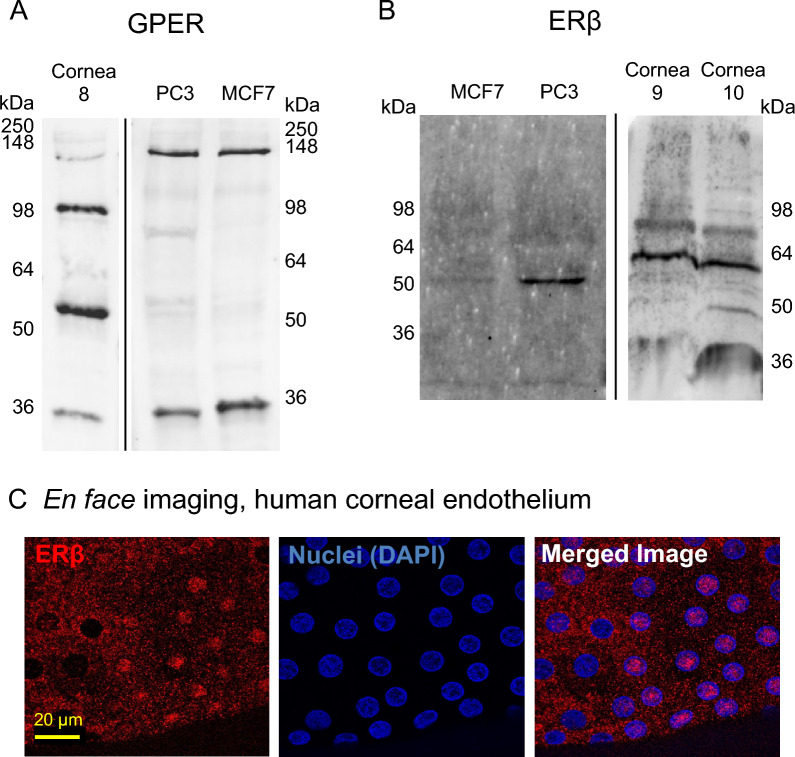
ER protein expression in human corneal endothelium. Western blotting was performed to measure protein levels of GPER (**A**) and ERβ (**B**) in native human corneal endothelium (*n* = 1 donor for GPER [cornea #8 in Table 1], *n* = 2 donors for ERβ [corneas #9 and 10]) and in the PC3 (*n* = 3 for each antibody) and MCF7 (*n* = 3 for each antibody) cell lines. Theoretical molecular weight: GPER, 42 kDa; ERβ, 55–60 kDa. The cropped blots from different gels are presented with dividing lines. Corresponding original images are provided in Supplementary Fig. 1. (**C**) *En face* immunofluorescence localization of ERβ in normal human corneal endothelium (representative images from corneas #11–13).

We further explored GPER and ERβ protein expression. The GPER antibody detected multiple distinct bands on Western blots of protein from human corneal endothelium, PC3 cells, and MCF-7 cells (Fig. 2A); however, none of the bands corresponded to the theoretical molecular weight of GPER (~ 42 kDa), suggesting alterative splicing of GPER transcripts, posttranslational modifications of GPER protein, or antibody detection of a similar antigen in different proteins. Differences in glycosylation of GPER are known to result in apparent differences in molecular weight on Western blot including ~ 37 and 124 kDa bands^26^, which are similar to those we observed in PC3 cells, MCF-7 cells, and human corneal endothelium (Fig. 2 and Supplementary Fig. 2). All major bands on the Western blot were blocked when the anti-GPER antibody was pre-incubated with the antigenic peptide used for antibody generation (Supplementary Fig. 2). We evaluated ERβ expression with two different antibodies. One antibody, rabbit anti-ERβ, detected a single band of expected molecular weight (55–60 kDa) in human retinal pigment epithelium, but detected two bands (~ 30 and 70 kDa) in human corneal endothelium and one prominent band (~ 65–70 kDa) in MCF-7 cells in which ERβ expression is anticipated to be low or absent (Supplementary Fig. 3)^27,28^. Because of these discrepancies in expression level and protein size, we thus tested a second ERβ antibody. The mouse anti-ERβ antibody detected a single protein at the expected molecular weight (55–60 kDa) in human corneal endothelium and, as expected, in PC3 cells^29^ but not in MCF-7 cells (Fig. 2B). ERβ was also present in native corneal endothelium, with cytoplasmic expression in most cells and nuclear expression in some (Fig. 2C). Although these data were limited in biological replicates to n = 2–3 donors and we did not have equal representation of male and female donor tissue, these data demonstrate that ERs are present in human corneal endothelium; thus, human corneal endothelium could be susceptible to receptor-mediated estrogen signaling.

### Effects of E2 and G1 on HCEnC growth

Estrogens can promote cell growth or cell death depending on the cell type or cell environment^30,31^. We studied the effect of E2 on primary cultures of HCEnCs. In addition, because GPER is the most abundant ER transcript in corneal endothelium, we studied the effects of the GPER agonist G1.

**Figure 3:**
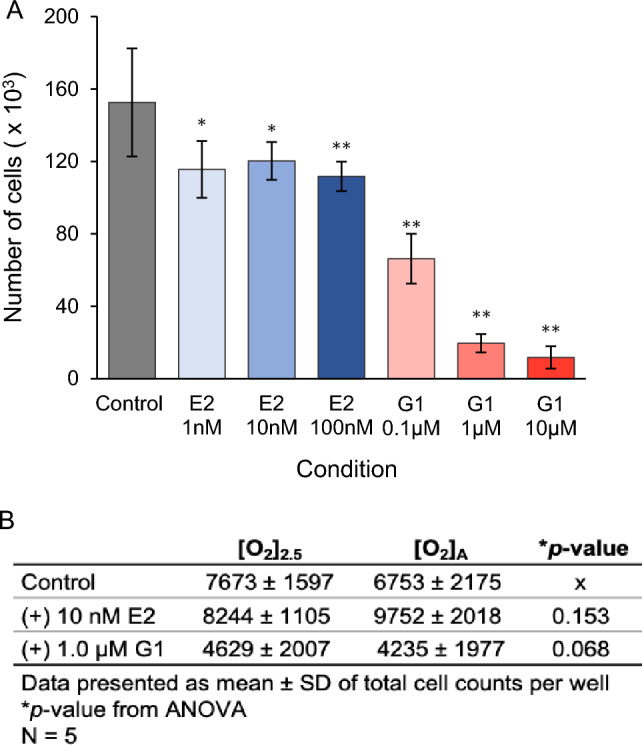
Effects of G1 and E2 on growth of PC3 cells and HCEnCs. (**A**) Counts of PC3 cells treated with 1 nM, 10 nM, or 100 nM E2 and 0.1 µM, 1.0 µM, or 10 µM G1 (*n* = 12). ∗ *p* < 0.01, ∗  ∗ *p* < 0.001 vs control by *t* test. (**B**) Counts of HCEnCs treated with 10 nM E2 or 1.0 μM G1 (*n* = 5, corneas #14–17 in Table 1). Data presented as means ± SDs of total cell counts per well. *p* values from single-factor ANOVA.

To establish the concentrations of G1 and E2 to use in experiments, we evaluated the effects of both drugs on PC3 cells. Treatment with E2 and G1 significantly decreased PC3 cell numbers (Fig. 3A). For the remainder of experiments described here, we used G1 at 1 μM and E2 at 10 nM.

We evaluated the effects of E2 and G1 on HCEnC growth. The HCEnCs in these experiments were cultured under physiologic O_2_ conditions^32^ with 2.5% O_2_ ([O_2_]_2.5_) and under the stress of chronic high oxygen conditions in a room air culture incubator ([O_2_]_A_)^22^. These two conditions were chosen to model the oxygen stress that occurs in FECD^8^. We found that, in contrast to the effects on PC3 cells, neither E2 nor G1 affected the growth of HCEnCs cultured under either O_2_ condition (Fig. 3B).

### Effects of E2 and G1 on HCEnC viability

**Figure 4:**
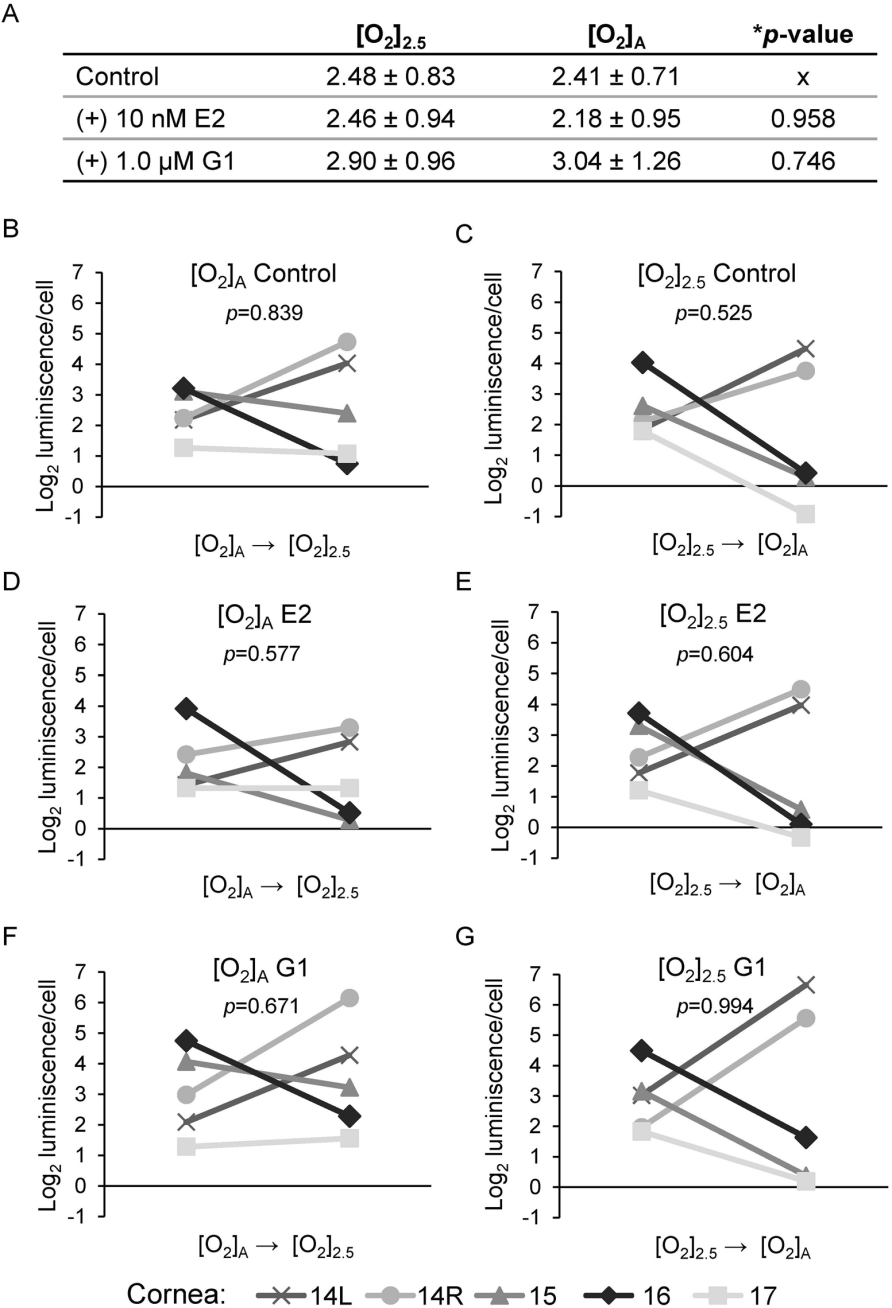
Viability of HCEnCs in the presence and absence of oxygen stress and E2 or G1 treatment. (**A**) Cell viability measurements (log_2_ luminescence/cell) for HCEnCs treated with E2 or G1 (*n* = 5, corneas #14–17 in Table 1). Data are presented as means ± SDs. *p value from ANOVA. Each plot in **B**–**G** represents the data for a single donor, with *p* values from paired *t* tests. Donor numbers correspond to those in Table 1.

Because E2 can affect more than just the growth of cells, we further investigated the effects of E2 on various functions of HCEnC. In particular, we were interested to see if E2 could affect the viability of HCEnCs in stressed environments and if G1 could reproduce the effects. We used the RealTime Glo MT cell viability assay to measure the reducing potential of HCEnCs in the presence and absence of oxygen stress, E2, and G1. This assay was chosen because it is a nonterminal assay in which measurements can be taken at multiple time points. Baseline measurements were taken after 4 days of incubation with E2 or G1 at either [O_2_]_A_ or [O_2_]_2.5_ (Fig. 4A). None of the measurements were significantly different from controls. We next evaluated if E2 or G1 affects viability upon the stress of reversal of the O_2_ culture condition. For this experiment, cultures at [O_2_]_A_ were placed at [O_2_]_2.5_, and cultures at [O_2_]_2.5_ were placed at [O_2_]_A_. The stress of the O_2_ condition change had variable effects on cells from different corneas (Fig. 4B–G), but there were no statistically significant differences in the response to stress under any condition.

### Oxidative stress

A key finding in FECD is that oxidative stress from oxidant-antioxidant imbalance leads to the accumulation of oxidized DNA lesions^7^. Furthermore, mitochondrial dysfunction and fragmentation from increased ROS are hallmarks of FECD that lead to corneal endothelial cell death^33,34^. In many cell types, oxidative stress is regulated by E2^35,36^. We therefore examined the role of E2 in mediating the effects of hyperoxic stress in HCEnCs by measuring ROS, changes to mitochondrial morphology, and oxidative DNA damage.

ROS levels in primary HCEnC cultures measured with an In Vitro ROS/RNS assay were unaffected by culture O_2_ conditions and E2 (Table 2). There were no differences in effects between samples from males and females.

**Table 2:** Levels of ROS in HCEnCs.

Condition	ROS (nM/µg protein)^a^		
	Male donor (*n* = 4)	Female donor (*n* = 5)	Total (*n* = 9)
[O_2_]_2.5_			
(−) E2	55.80 ± 32.99	19.45 ± 6.68	40.22 ± 30.60
(+) E2	32.62 ± 15.34	28.31 ± 9.72	30.22 ± 11.86
[O_2_]_A_			
(−) E2	24.70 ± 15.15	23.94 ± 17.57	24.45 ± 14.13
(+) E2	43.44 ± 34.43	31.44 ± 8.56	36.77 ± 22.83
*p* value^b^	0.42	0.4	0.53

^a^Data are means ± SDs. Corneas #18–26L in Table 1.

^b^*p* values from ANOVA.

**Figure 5:**
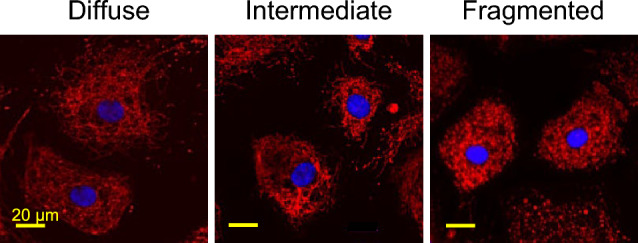
Representative images of mitochondrial morphology grading. Mitochondrial morphology revealed by MitoTracker Red was graded for each cell as diffuse (normal), intermediate, or fragmented (abnormal). Nuclei were stained with DAPI (blue). Representative images from corneas #27R–34L in Table 1.

**Figure 6:**
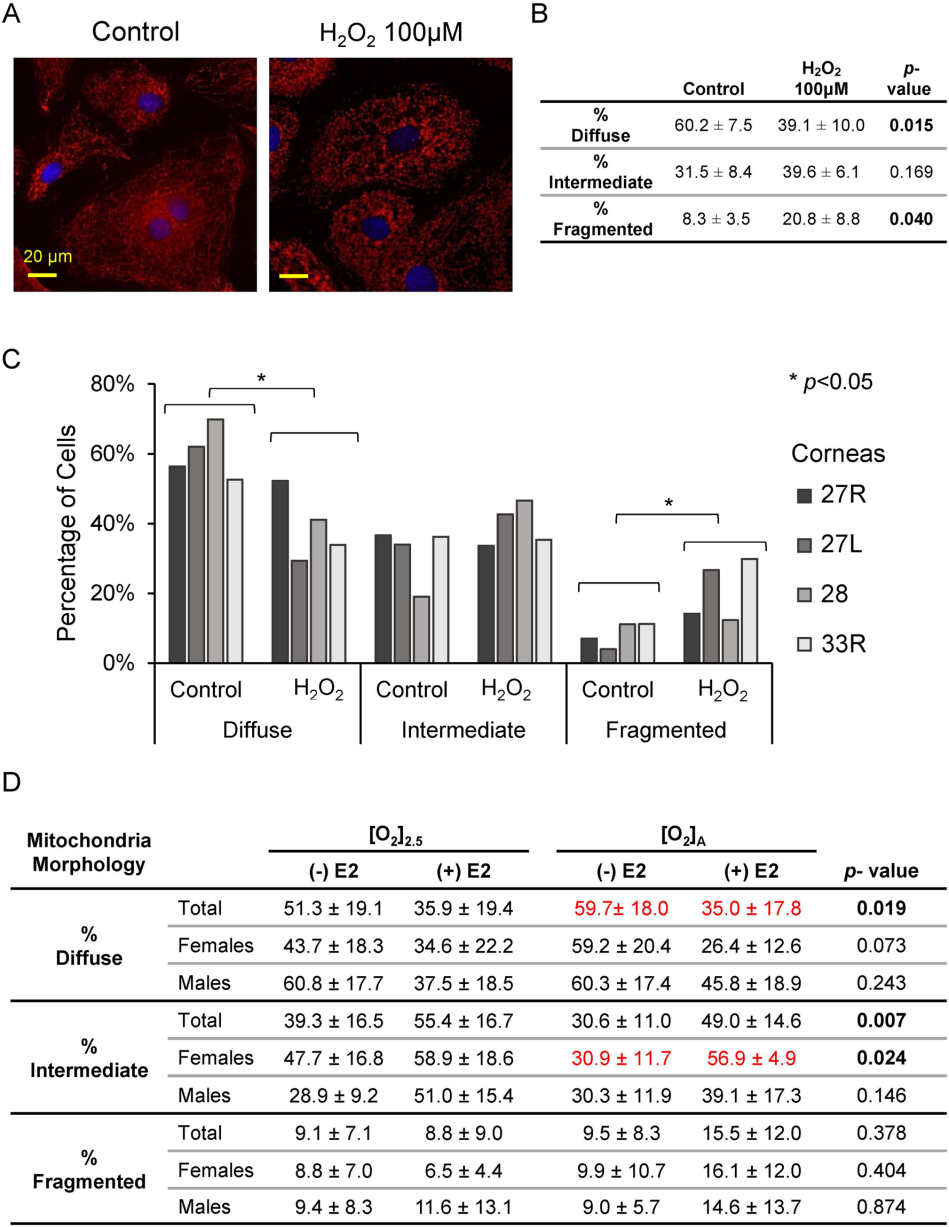
Mitochondrial morphology in HCEnCs in the presence and absence of oxygen stress and E2 treatment. (**A**) Immunofluorescence images of mitochondria in HCEnCs stained with MitoTracker Red after H_2_O_2_ treatment. (**B**, **C**) Quantification of mitochondrial organization graded as diffuse (normal), intermediate, or fragmented (abnormal) in HCEnCs treated with H_2_O_2_ (*n* = 4, corneas #27R, 27L, 28L, and 33R in Table 1). *p* values from *t* tests. Data presented as means ± SDs. (**D**) Quantification of mitochondrial organization graded in HCEnCs in the presence or absence of 10 nM E2 at [O_2_]_2.5_ or [O_2_]_A_. *p* value from single-factor ANOVA. Pairwise comparisons by Tukey's post hoc analysis, with significant differences highlighted in red. (*n* = 9 [4 males and 5 females]; corneas #27R–33L).

We next evaluated qualitative changes in mitochondrial morphology. Mitochondria were stained with MitoTracker Red CMXRos, and the mitochondrial phenotypes were classified in each cell as diffuse (normal), fragmented (abnormal), or intermediate (Fig. 5)^25^. A positive control with 100 μM H_2_O_2_ showed an expected significant decrease in the percentage of cells with diffuse mitochondrial organization and an increase in the percentage of cells with fragmented mitochondrial organization (Fig. 6A–C). When the HCEnCs were exposed to [O_2_]_A_ stress in the presence or absence of E2, we found a significant decrease (*p* = 0.019, single factor ANOVA) in the percentage of cells with diffuse mitochondrial organization and an increase in cells with intermediate mitochondrial organization (*p* = 0.007; Fig. 6D). A post hoc analysis revealed that the significant decrease in cells with diffuse mitochondria was only at [O_2_]_A_ with 10 nM E2 compared to that at [O_2_]_A_ without E2 (*p* = 0.039). The post hoc tests for intermediate morphology for the total experimental population revealed no physiologically relevant differences. However, when the intermediate morphology data were divided by the sex of the corneal donor tissue, there was a significant increase in the percentage of cells with intermediate mitochondrial morphology in females at [O_2_]_A_ with 10 nM E2 compared to that at [O_2_]_A_ without E2 (*p* = 0.044). There were no significant differences in males. There were no differences in mitochondrial morphology due to the hyperoxic stress alone. These data show that detrimental changes to mitochondrial morphology in HCEnCs occur in response to hyperoxic stress only in the presence of E2 in females.

**Figure 7:**
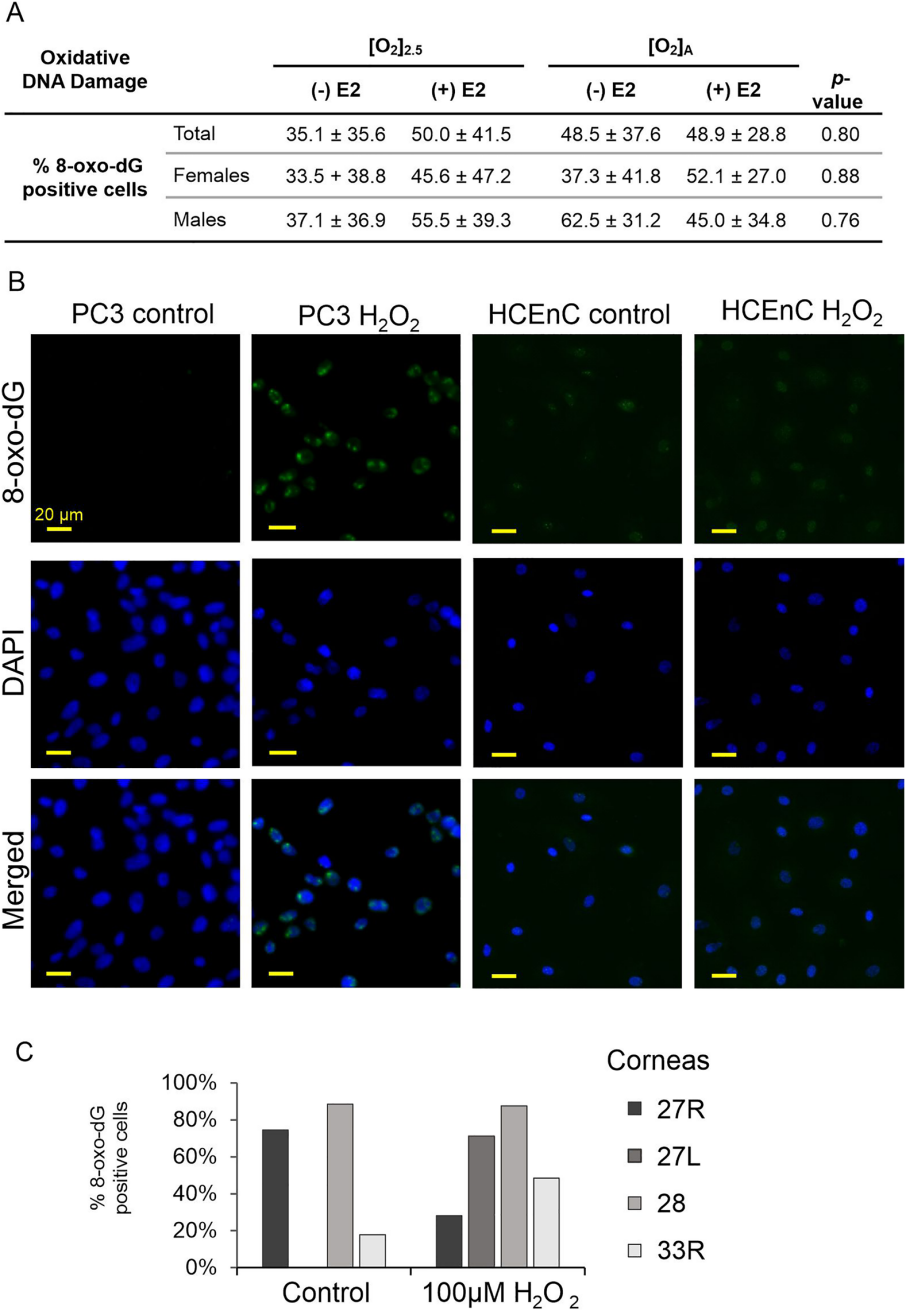
Oxidative damage in HCEnCs in the presence and absence of oxygen stress and E2 treatment. (**A**) Percentages of 8-oxo-dG-positive HCEnCs in the presence or absence of 10 nM E2 at [O_2_]_2.5_ or [O_2_]_A_. Data presented as means ± SDs. *p* value from single-factor ANOVA (*n* = 8 [4 males and 4 females]; corneas #27L–33L in Table 1). (**B**) Immunofluorescence imaging of 8-oxo-dG in PC3 cells and HCEnCs after H_2_O_2_ treatment. (**C**) Percentages of 8-oxo-dG-positive HCEnCs cells after H_2_O_2_ treatment (*n* = 4, corneas #27R, 27L, 28L, and 33R).

Mitochondrial stress can also result in cellular oxidative stress with oxidative DNA damage. We tested for the oxidative DNA damage marker 8-oxo-dG in HCEnCs in response to O_2_ and E2. We found no significant differences in the 8-oxo-dG signals in HCEnCs under any condition (*p* < 0.05, single factor ANOVA; *n* = 8 [4 females, 4 males]) (Fig. 7A). Likewise, there were no significant differences when data were separated by donor sex. Interestingly, there was also no significant increase in 8-oxo-dG in HCEnCs exposed to 100 μM H_2_O_2_ for 48 h. However, treatment with 100 μM H_2_O_2_ for 48 h lead to complete death of PC3 cells; treatment for 6 h dramatically increased nuclear 8-oxo-dG signals, demonstrating that the treatment induced oxidative stress (Fig. 7B,C).

### Cellular energetics

The observed changes in mitochondrial morphology with E2 prompted us to investigate if E2 also affects HCEnC metabolism. We measured changes in total cellular ATP levels and also evaluated for changes in oxidative respiration and glycolysis.

Cellular ATP levels were significantly different amongst the four conditions analyzed ([O_2_]_A_ ± E2 and [O_2_]_2.5_ ± E2; ANOVA *p* = 0.01; *n* = 7–9) (Table 3). A post hoc analysis showed significant differences in ATP levels between cells grown at [O_2_]_A_ and those grown at [O_2_]_2.5_ without E2 (*p* = 0.02). There were no significant effects of 10 nM E2 addition. However, there was a 1.8-fold difference in ATP levels between cells with E2 treatment those without E2 at [O_2_]_2.5_ (*p* = 0.07). When the data were segregated by sex of the donors, a cell sex-specific pattern was noted. ATP levels in corneas from females showed significant differences among the conditions (ANOVA *p* = 0.01), while levels in corneas from males did not (ANOVA *p* = 0.77). ATP levels in HCEnCs from female donors were higher at [O_2_]_2.5_ than at [O_2_]_A_ (*p* = 0.01) in the absence of E2.

**Table 3:** ATP levels in HCEnCs.

Condition	ATP (μM)^a^		
	Male donor (*n* = 4)	Female donor (*n* = 5)	Total (*n* = 9)
[O_2_]_2.5_			
(−) E2	3.85 ± 1.29	**4.83 ± 1.33**	**4.46 ± 1.32**
(+) E2	3.72 ± 1.38	2.75 ± 1.64	3.30 ± 1.45
[O_2_]_A_			
(−) E2	3.22 ± 0.61	**2.37 ± 0.30**	**2.74 ± 0.62**
(+) E2	3.07 ± 1.28	2.22 ± 0.35	2.71 ± 1.03
*p* value^b^	0.77	**0.009**	**0.01**

^a^Data are means ± SDs. Corneas #26R, 35R–39L in Table 1.

^b^*p* values from ANOVA with Tukey's post hoc analysis. Significant differences are in bold font.

**Figure 8:**
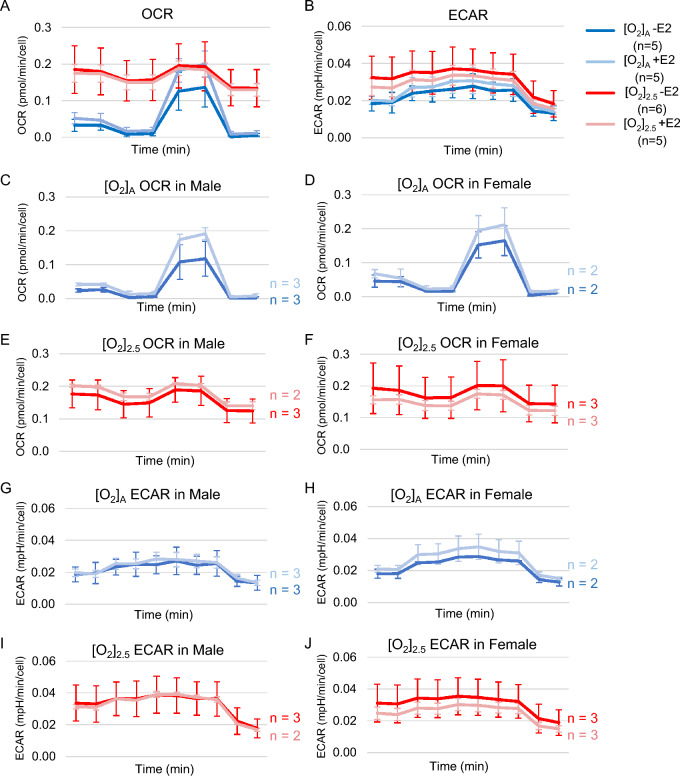
OCR and ECAR in the presence and absence of oxygen stress and E2 treatment. OCR (**A**) and ECAR (**B**) measurements in the presence or absence of 10 nM E2 at [O_2_]_2.5_ or [O_2_]_A_. (**C**–**J**) Trends in mean data values with and without E2 treatment at [O_2_]_2.5_ and [O_2_]_A_ by cell sex for each condition. *x* axis for time is a total duration of 41 min for OCR and 52 min for ECAR recordings. Data presented as means ± SDs (*n* = 5–6 [2–3 males and 2–3 females]; corneas #40R–45 in Table 1).

To further characterize the effects of E2 on the energetics of HCEnC metabolism, we measured OCR for oxidative respiration and ECAR for glycolysis at [O_2_]_A_ and [O_2_]_2.5_ with and without E2. [O_2_]_2.5_ with and without E2 resulted in higher OCR and ECAR measurements that those at [O_2_]_A_; however, there were no statistically significant differences in OCR or ECAR due to E2 at [O_2_]_A_ or [O_2_]_2.5_ (Fig. 8A,B).

Because of our small sample size (*n* = 2–3 males and 2–3 females), we did not have statistical power sufficient to identify significant differences in E2 effects by sex. However, the data trends for E2 effects by sex for each O_2_ condition are plotted in Fig. 8C–J.

## Discussion

Risk factors for FECD include older age and smoking, and both factors are associated with increased oxidative stress^4,15^. Menopause in women is also associated with increased oxidative stress from decreased levels of estrogen post-menopause^37^. The higher prevalence of FECD in women than in men thus prompted a search for the role of estrogen in FECD. Prior in vitro work indicated that estrogen metabolites are toxic to corneal endothelium^16^. Yet, for other chronic degenerative diseases, including ocular diseases such as macular degeneration^38,39^ and glaucoma^40–42^, estrogen shows beneficial effects. We explored the role of estrogen and ER-mediated effects in human corneal endothelium.

Prior data on ER expression in corneal endothelium have been sparse and contradictory^43–45^. We observed expression of ERβ and GPER, but not ERα, in HCEnCs, thus confirming that ERs are present in corneal endothelium. ERs have antagonistic effects in many diseases, especially cancers, with ERα most commonly promoting cell proliferation and ERβ and GPER inhibiting tumor growth^19^. We found that E2 and G1 inhibited the growth of prostate cancer (PC3) cells, as might be expected for cells expressing ERβ and GPER; however, there were no statistically significant effects on growth of HCEnCs. We used primary HCEnCs from older human donors, and it is possible that the growth potential of the cells was limited; nevertheless, we anticipated seeing a robust inhibition of growth if indeed it were present.

ERβ and GPER also have significant roles in regulating cell metabolism, and estrogens mitigate the damaging effects of oxidative stress^18,35,46^. ERβ is the primary ER found in mitochondria, and multiple GPER-targeted interventions (pharmacologic and genetic) affect cell metabolism^47–50^. Dysregulated cell energetics, mitochondrial dysfunction, and oxidative stress are hallmarks of FECD^34,51,52^. We therefore measured oxidative stress, mitochondrial dysfunction, and cell metabolism in HCEnCs under physiologic oxygen conditions ([O_2_]_2.5_) and under hyperoxic stress ([O_2_]_A_) in the presence and absence of E2. We were intrigued to find that the primary HCEnCs had stable levels of ROS and were resistant to oxidative DNA damage, even under conditions such as 100 μM H_2_O_2_ that killed PC3 cells. This is similar to the observations of others that HCEnCs in primary cultures can restore oxidant-antioxidant balance and resist oxidative stress^53^ but in contrast to the findings in which HCEnCs are readily affected by oxidative stress^54^.

Despite the absence of differences in our measures of mitochondrial morphology with hyperoxic stress in HCEnCs, we detected significant effects of E2, but only for cells derived from female donors. These data support a cell sex-specific detrimental effect of E2 when HCEnCs are exposed to hyperoxic stress. The interaction of E2 and cell sex may contribute to development of the sex disparity noted in FECD prevalence. It is likely that multiple stressors are required over time to generate the FECD phenotype^33^.

There may also be cell sex-dependent but E2-independent differences in HCEnC energetics because we found different concentrations of ATP in female HCEnCs but not male HCEnCs at [O_2_]_A_ versus [O_2_]_2.5_. It is presently unclear whether to interpret the higher ATP levels in female HCEnCs at [O_2_]_2.5_ than at [O_2_]_A_ as favorable or unfavorable. Because compensatory increases in mitochondrial density precede mitochondrial burnout in HCEnCs^33^, it is possible that female HCEnCs at [O_2_]_2.5_ are sick whereas male cells or cells under the [O_2_]_A_ condition are not. However, overall, we observed more favorable growth of HCEnCs at [O_2_]_2.5_ than at [O_2_]_A_, suggesting that the cells were not sick^22^. Our data do not resolve this issue but do confirm that cell sex influences HCEnC behavior. Data from other cell types also support the presence of estrogen-independent mechanisms for cell sex differences in cell behavior^36,55^.

Through these studies, we have shown that ERβ and GPER are present in HCEnCs, thus providing the necessary framework for receptor-mediated estrogen signaling in HCEnCs. Furthermore, we showed that mitochondria and energetics of HCEnCs have estrogen-dependent and cell sex-dependent effects according to the O_2_ culture environment. We conclude that estrogen signaling and/or cell sex contribute to HCEnC function and dysfunction in an environment-specific fashion.

### Supplementary Information

Supplementary Figures.

Supplementary Table 1.

## Data Availability

The datasets generated during and/or analyzed during the current study are available from the corresponding author on reasonable request.
