# Supplementary material for: Selective effects of estradiol on human corneal endothelial cells
Source: Sci Rep. 2023 Sep 15;13:15279. doi: 10.1038/s41598-023-42290-z (PMC10504266; doi:10.1038/s41598-023-42290-z)

Supplementary Figure 1. Uncropped Western blot images for manuscript Figure 2. 10 µg protein loaded per lane. MWM = Molecular Weight Marker. Imaged with ChemiDoc MP Imaging System.

GPER in PC3 and MCF-7 cell lines

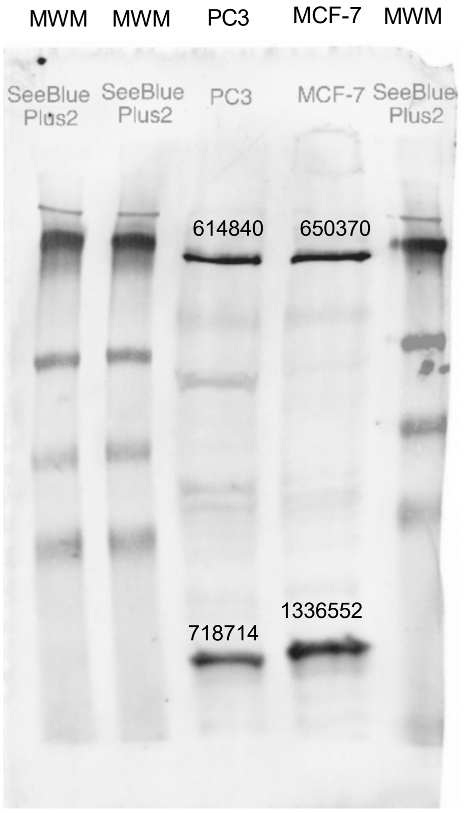

GPER in native human corneal endothelium

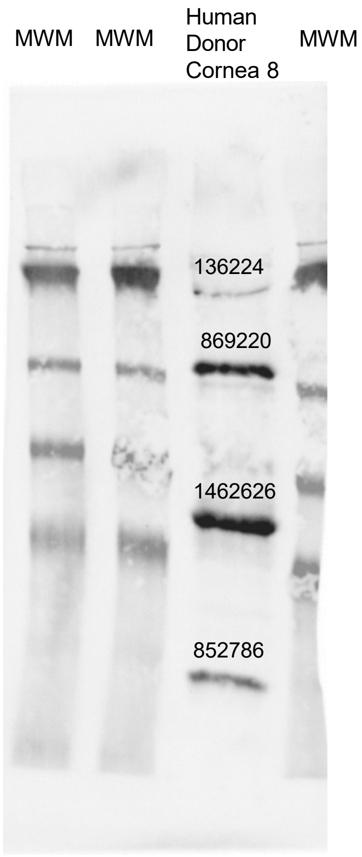

ERβ in PC3 and MCF-7 cell lines

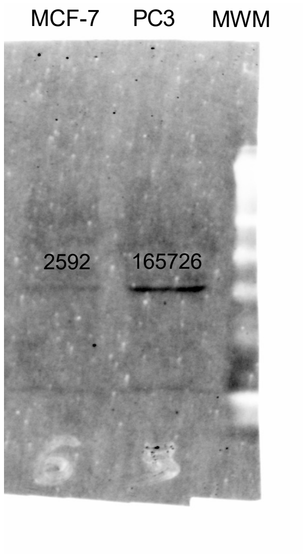

ERβ in native human corneal endothelium

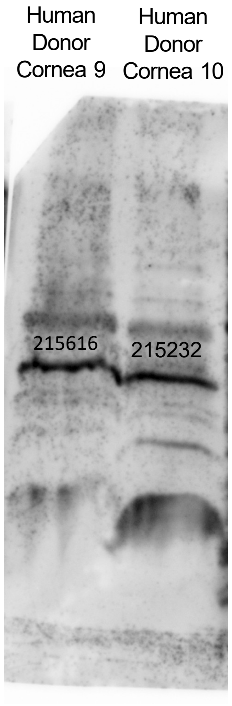

mouse anti-ERβ antibody: cat# sc-390243, lot# K1814; Santa Cruz Biotechnology

Band intensity values are indicated in the vicinity of each main band. The Lane Profile tools of Image Lab software version 6.0.1 (Bio-Rad Laboratories) were used to select and determine the background-subtracted intensity of the bands.

Supplementary Figure 2. Uncropped Western blot images for GPER expression in the presence and absence of blocking peptide. Rabbit anti-GPER1 antibody (cat# HPA027052, lot# D118286 [Sigma-Aldrich]) was applied to the blot with and without a 45 minute pre-incubation with a 100 molar excess of the corresponding antigenic peptide (PrEST Antigen GPER1, cat# APrEST72764, lot# PRL01683, [Sigma-Aldrich]). 10 µg protein loaded per lane. MWM = Molecular Weight Marker. Imaged with ChemiDoc MP Imaging System.

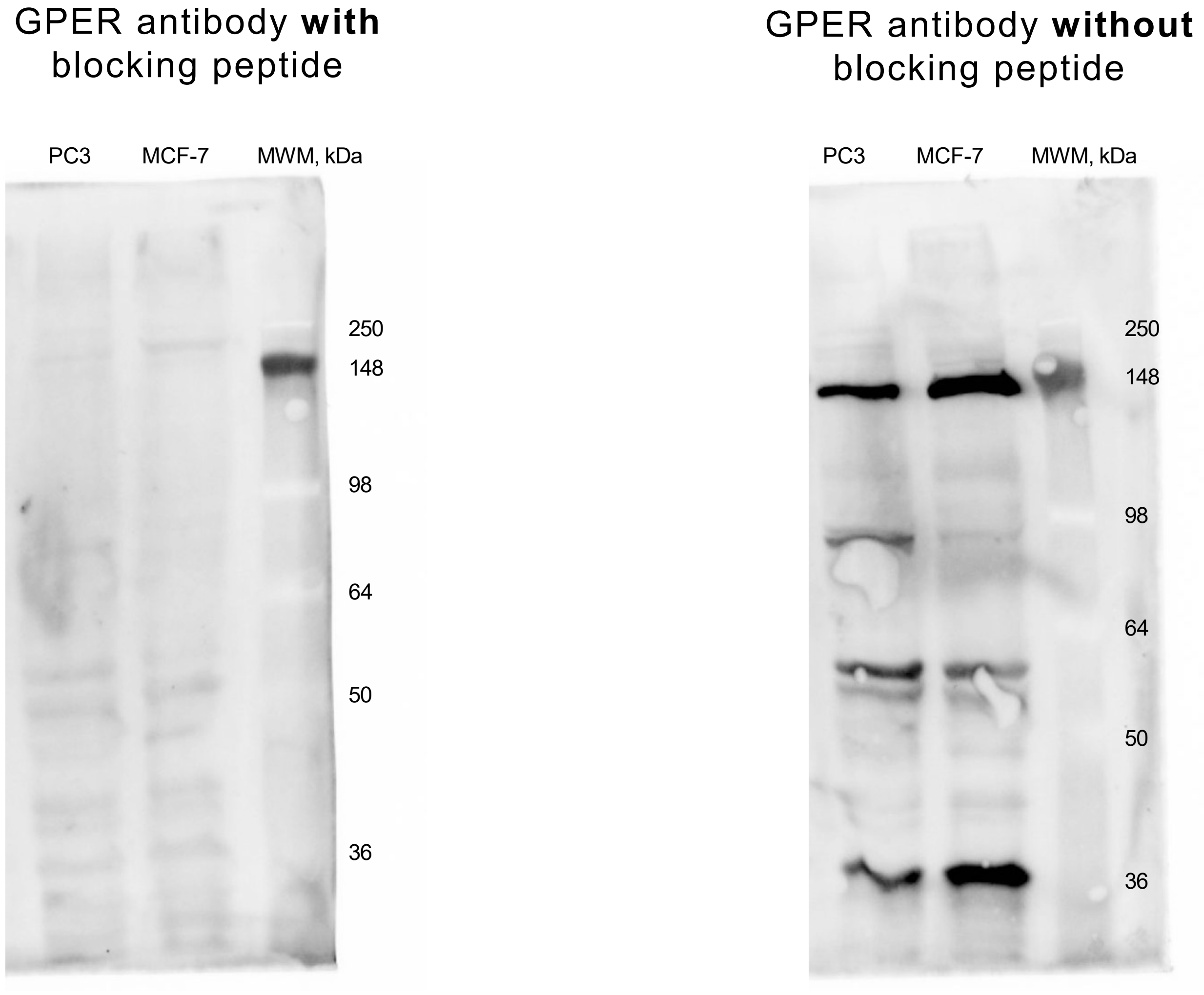

Supplementary Figure 3. Uncropped Western blot images for ERβ expression (sc-8974, Lot # D0615, rabbit polyclonal ERβ antibody, Santa Cruz Biotechnology; 1:1000 dilution). This is a different antibody than used for manuscript Figure 2 and Supplementary Figure 1. 10 μg protein loaded per lane. MWM = Molecular Weight Marker. Imaged with ChemiDoc MP Imaging System.

ERβ in human retinal pigment epithelium (RPE) and native human corneal endothelium

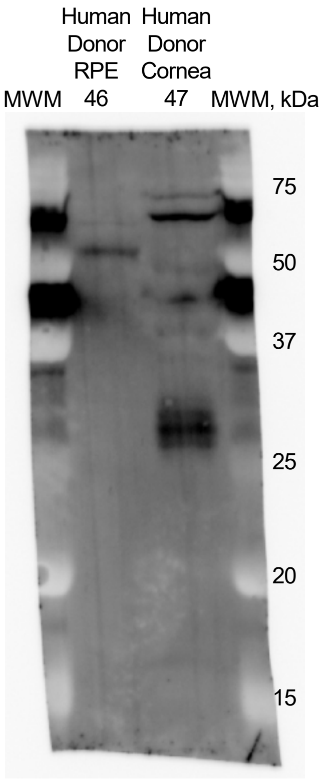

ERβ in MCF-7 cells and native human corneal endothelium

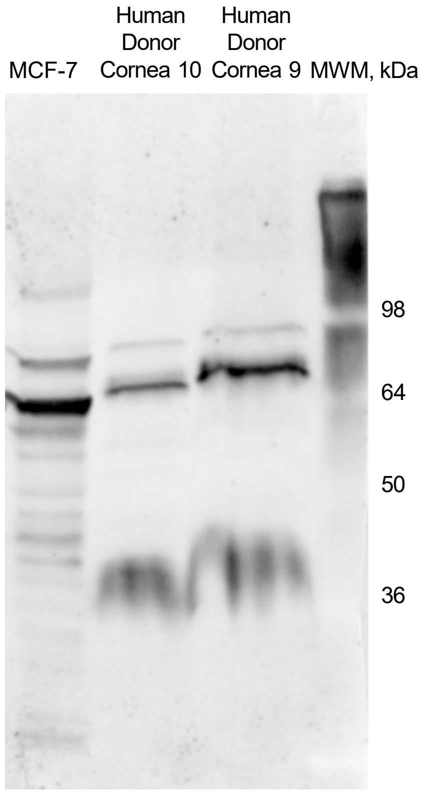

Supplement: Supplementary file 1 — Supplementary Figures. [file 41598_2023_42290_MOESM1_ESM.pdf]
