# Supplementary material for: Selective effects of estradiol on human corneal endothelial cells
Source: Sci Rep. 2023 Sep 15;13:15279. doi: 10.1038/s41598-023-42290-z (PMC10504266; doi:10.1038/s41598-023-42290-z)
Supplement: Supplementary file 2 — Supplementary Table 1. [file 41598_2023_42290_MOESM2_ESM.pdf]

Supplementary Table 1. Primer list for quantitative RT-PCR.

Gene	Protein	BioRad Unique Assay ID
ER1α	estrogen receptor 1	qHsaCED0042641
ER2β	estrogen receptor 2	qHsaCED0044944
GPER	G protein-coupled estrogen receptor 1	qHsaCED0021538
AQP1	aquaporin 1 (Colton blood group)	qHsaCED0048762
GPDH	glyceraldehyde-3-phosphate dehydrogenase	qHsaCED0038674
CANX	calnexin	qHsaCID0010564
LDHA	lactate dehydrogenase A	qHsaCED0056429
RPLP0	ribosomal protein, large, P0	qHsaCED0038653
UBC	ubiquitin C	qHsaCED0023867
